# Supplementary material for: Heat adaptation of phage T7 under an extended genetic code
Source: Virus Evol. 2021 Dec 1;7(2):veab100. doi: 10.1093/ve/veab100 (PMC8923235; doi:10.1093/ve/veab100)
Supplement: veab100_Supp [file veab100_supp.zip › Sequencing_Analysis.zip!/Sequencing_Analysis/A2/evidence/JC_45.html]

BRESEQ :: Evidence


| New junction evidence | | | | | | | | | | | |
| --- | --- | --- | --- | --- | --- | --- | --- | --- | --- | --- | --- |
|  | | seq id | position | reads (cov) | reads (cov) | score | skew | freq | annotation | gene | product |
| \* | ? | NC\_001604.1 | = 7605 | 543 (1.190) | 8 (0.020) | 3/578 | 23.3 | 1.5% | intergenic (–/–) | *–/–* | –/– |
|  | ? | NC\_001604.1 | 7904 = | 504 (1.150) |  |  |  |  | intergenic (–/–) | *–/–* | –/– |
| Rejected: Coverage evenness skew score above cutoff. | | | | | | | | | | | |
| Rejected: Frequency below/above cutoff threshold. | | | | | | | | | | | |

`GTGGTCGTTTAGTTAACGCCACGAATATCTCTCGCGCCTTAATGGATGAGTTCACTGAGACAGTAAAAGAGGCCACCCTAAGTCAATGGGGATTCTTTAGCCCATACGGTATTGGCGACAACGATGCTTGTACTATTAACCCTTACGATGGCTGGGCGTGTCAAATTAGCTACATGGAGGAAACACCTGATGGCTCTTTGCGGCACCCATCGTTCGTAATGTTCCGTGGCACCGAGGACAACCCTCAAGAGAAAATGTAATCACACTGGCTCACCTTCGGGTGGGCCTTTCTGCGTTTATAAGGAGACACT‑‑‑‑‑‑‑‑‑‑‑‑‑‑‑‑‑‑‑‑‑‑‑‑‑‑‑‑‑‑‑‑‑‑‑‑‑‑‑‑‑‑‑‑‑‑‑‑‑‑‑‑‑‑‑‑‑‑‑‑‑‑‑‑‑‑‑‑‑‑‑‑‑‑‑‑‑‑‑‑‑‑‑‑‑‑‑‑‑‑‑‑‑‑‑‑‑‑‑‑‑‑‑‑‑‑‑‑‑‑‑‑‑‑‑‑‑‑‑‑‑‑‑‑‑‑‑‑‑‑‑‑‑‑‑‑‑‑‑‑‑‑‑‑‑‑‑‑‑‑‑‑‑‑‑‑‑‑‑‑‑‑‑‑‑‑‑‑‑‑‑‑‑‑‑‑‑‑‑‑‑‑‑‑‑‑‑‑‑‑‑‑‑‑‑‑‑‑‑‑‑‑‑‑‑‑‑‑‑‑‑‑‑‑‑‑‑‑‑‑‑‑‑‑‑‑‑‑‑‑‑‑‑‑‑‑‑‑‑‑‑‑‑‑‑‑‑‑‑‑‑‑‑‑‑‑‑‑‑‑‑‑‑‑‑‑‑‑‑‑‑‑‑‑‑‑‑‑‑‑‑‑‑‑‑‑‑‑‑‑‑‑‑‑‑‑‑‑‑  >  NC_001604.1/7295‑7605`  
`‑‑‑‑‑‑‑‑‑‑‑‑‑‑‑‑‑‑‑‑‑‑‑‑‑‑‑‑‑‑‑‑‑‑‑‑‑‑‑‑‑‑‑‑‑‑‑‑‑‑‑‑‑‑‑‑‑‑‑‑‑‑‑‑‑‑‑‑‑‑‑‑‑‑‑‑‑‑‑‑‑‑‑‑‑‑‑‑‑‑‑‑‑‑‑‑‑‑‑‑‑‑‑‑‑‑‑‑‑‑‑‑‑‑‑‑‑‑‑‑‑‑‑‑‑‑‑‑‑‑‑‑‑‑‑‑‑‑‑‑‑‑‑‑‑‑‑‑‑‑‑‑‑‑‑‑‑‑‑‑‑‑‑‑‑‑‑‑‑‑‑‑‑‑‑‑‑‑‑‑‑‑‑‑‑‑‑‑‑‑‑‑‑‑‑‑‑‑‑‑‑‑‑‑‑‑‑‑‑‑‑‑‑‑‑‑‑‑‑‑‑‑‑‑‑‑‑‑‑‑‑‑‑‑‑‑‑‑‑‑‑‑‑‑‑‑‑‑‑‑‑‑‑‑‑‑‑‑‑‑‑‑‑‑‑‑‑‑‑‑‑‑‑‑‑‑‑‑‑‑‑‑‑‑‑‑‑‑‑‑‑‑‑‑‑‑‑‑‑‑aaggagacactATATGTTTCGACTTCATTACAACAAAAGCGTTAAGAATTTCACGGTTCGCCGTGCTGACCGTTCAATCGTATGTGCGAGCGAGCGCCGAGCTAAGATACCTCTTATTGGTAACACAGTTCCTTTGGCACCGAGCGTCCACATCATTATCACCCGTGGTGACTTTGAGAAAGCAATAGACAAGAAACGTCCGGTTCTTAGTGTGGCAGTGACCCGCTTCCCGTTCGTCCGTCTGTTACTCAAACGAATCAAGGAGGTGTTCTGATGGGACTGTTAGATGGTGAAGCCTGGGAAAAAGAAA  >  NC_001604.1/7904‑8202`  

                                                                                                                                                                                                                                                                                                                                                                                                                                                                                                                                                                                                                                    
`gacagCGTTTAGTTAACGCCACTAATATCTCTCGCGCCTTAATGGATGAGTTCACTGAGACAGTAAAAGAGGCCACCCTAAGTCAATGGGGATTCTTTAGCCCATACGGTATTGGCGACAACTATGCTTGTACTATTAACCCTTACGATGGCTGGGCGTGTCAAATTAGCTACATGGAGGAAACACCTTATGGCTCTTTTCGGCACCCATCGTTCGTAATGTTCCGTGGCACCGAGGACAACCCTCAAGAGAAAATGTAATCACACTGGCTCACCTTCGGGTGGGCCTTTCTGCGTTTATA                                                                                                                                                                                                                                                                                                                       <  1:390290/296‑1`  
  `GGTCGTTTAGTTAACGCCACGAATATCTCTCGCGCCTTAATGGATGAGTTCACTGAGACAGTAAAAGAGGCCACCCTAAGTCAATGGGGATTCTTTAGCCCATACGGTATTGGCGACAACGATGCTTGTACTATTAACCCTTACGATGGCTGGGCGTGTCAAATTAGCTACATGGAGGAAACACCTGATGGCTCTTTGCGGCACCCATCGTTCGTAATGTTCCGTGGCACCGAGGACAACCCTCAAGAGAAAATGTAATCACACTGTCTCACCTTCGGGTGGGCCTTTCTGCGTTTATAAG                                                                                                                                                                                                                                                                                                                     <  1:626760/301‑1`  
   `agatgtgtataagagacagGAATATCTCTCGCGCCTTAATGGATGAGTTCACTGAGACAGTAAAAGAGGCCACCCTAAGTCAATGGGGATTCTTTAGCCCATACGGTATTGGCGACAACGATGCTTGTACTATTAACCCTTACGATGGCTGGGCGTGTCAAATTAGCTACATGGAGGAAACACCTGATGGCTCTTTGCGGCACCCATCGTTCGTAATGTTCCGTGGCACCGAGGACAACCATCAAGAGAAAATGTAATCACACTGGCTCACCTTCGGGTGGGCCTTTCTGCGTTTATAAGG                                                                                                                                                                                                                                                                                                                    <  1:779503/282‑1`  
   `GTCGTTTAGTTAACGCCACGAATATCTCTCGCGCCTTAATGGATGAGTTCACTGAGACAGTAAAAGAGGCCACCCTAAGTCAATGGGGATTCTTTAGCCCATACGGTATTGGCGACAACGATGCTTGTACTATTAACCCTTACGATGGCTGGGCGTGTCAAATTAGCTACATGGAGGAAACACCTGATGGCTCTTTGCGGCACCCATCGTTCGTAATGTTCCGTGGCACCGAGGACAACCCTCACGAGAAAATGTAATCACACTGGCTCACCTTCGGGTGGGCCTTTCTGCGTTTATAAGG                                                                                                                                                                                                                                                                                                                    >  1:276092/1‑301`  
   `GTCGTTTAGTTAACGCCACGAATATCTCTCGCGCCTTAATGGATGAGTTCACTGAGACAGTAAAAGAGGCCACCCTAAGTCAATGGGGATTCTTTAGCCCATACGGTATTGGCGACAACGATGCTTGTACTATTAACCCTTACGATGGCTGGGCGTGTCAAATTAGCTACATGGAGGAAACACCTGATGGCTCTTTGCGGCACCCATCGTTCGTAATGTTCCGTGGCACCGAGGACAACCCTCAAGAGAAAATGTAATCACACTGGCTCACCTTCGGGTGGGCCTTTCTGCGTTTATAAGG                                                                                                                                                                                                                                                                                                                    <  1:182641/301‑1`  
     `CGTTTAGTTAACGCCACGAATATCTCTCGCGCCTTAATGTATGAGTTCACTGAGACAGTAAAAGAGGCCACCCTAAGTCAATGGGGATTCTTTAGCCCATACGGTATTGGCGACAACGATGCTTGTACTATTAACCCTTACGATGGCTGGGCGTGTCAAATTAGCTACATGGATGAAACACCTGATGGCTCTTTGCGGCACCCATCGTTCGTAATGTTCCGTGGCACCGAGGACAACCCTCAAGAGAAAATGTAATCACACTGGCTCACCTTCGGGTGGGCCTTTCTGCGTTTATAAGGAG                                                                                                                                                                                                                                                                                                                  >  1:419246/1‑301`  
     `CGTTTAGTTAACGCCACGAATATCTCTCGCGCCTTAATGGATGAGTTCACTGAGACAGTAAAAGAGGCCACCCTAAGTCAATGGGGATTCTTTAGCCCATACGGTATTGGCGACAACGATGCTTGTACTATTAACCCTTACGATGGCTGGGCGTGTCAAATTAGCTACATGGAGGAAACACCTGATGGCTCTTTGCGGCACCCATCGTTCGTAATGTTCCGTGGCACCGAGGACAACCATCAAGAGAAAATGTAATCACACTGGATCACCTTCGGGTGGGCCTTTCTGCGTTTGTAAGGAG                                                                                                                                                                                                                                                                                                                  >  1:592367/1‑301`  
       `gggctcggagatgtgtataagagacaGCGCCTTAATGGATGAGTTCACTGAGACAGTAAAAGAGGCCACCCTAAGTCAATGGGGATTCTTTAGCCCATACGGTATTGGCGACAACGATGCTTGTACTATTAACCCTTACGATGGCTGGGCGTGTCAAATTAGCTACATGGAGGAAACACCTGATGGCTCTTTGCGGCACCCATCGTTCGTAATGTTCCGTGGCACCGAGTACAACCCTCAAGAGAAAATGTAATCACACTGGCTCACCTTCGGGTGGGCCTTTCTGCGTTTATAAGGAGAt                                                                                                                                                                                                                                                                                                                <  1:8585/275‑2`  
         `ataagagacagACGAATATCTCTCGCGCCTTAATGGATGAGTTCACTGAGACAGTAAAAGAGGCCACCCTAAGTCAATGGGGATTCTTTAGCCCATACGGTATTGGCGACAACGATGCTTGTACTATTAACCCTTACGATGGCTGGGCGTGTCAAATTAGCTACATGGAGGAAACACCTGATGGCTCTTTGCGGCACCCATCGTTCGTAATGTTCCGTGGCACCGAGGACAACCCTCAAGAGAAAATGTAATCACACTGGCTCACCTTCGGGTGGGCCTTTCTGCGTTTATAAGGAGACAC                                                                                                                                                                                                                                                                                                              <  1:578197/290‑1`  
                           `TCTCTCGCGCCTTAATGGATGAGTTCACTGAGACAGTAAAAGAGGCCACCCTAAGTCAATGGGGATTCTTTAGCCCATACGGTATTGGCGACAACGATGCTTGTACTATTAACCCTTACGATGGCTGGGCGTGTCAAATTAGCTACATGGAGGAAACACCTGATGGCTCTTTGCGGCACCCATCGTTCGTAATGTTCCGTGGCACCGAGGACAACCCTCAAGAGAAAATGTAATCACACTGGCTCACCTTCGGGTGGGCCTTTCTGCGTTTATAAGGAGctgtctcttatacccatctccg                                                                                                                                                                                                                                                                                            >  1:383839/1‑279`  
                                         `ATGGATGAGTTCACTGCGACAGTAAAAGAGGCCACCCTAAGTCAATGTGGATTCTTTAGCCCATACGGTATTGGCGACAACGATGCTTGTACTATTAACCCTTACGATGGCTGGGCGTGTCAAATTAGCTACATGGAGGAAACACCTGATGGCTCTTTGCGGCACCCATCGTTCGTAATGTTCCGTGGCACCGAGGACAACCCTCAAGAGAAAATGTAATCACACTGGCTCACCTTCGGGTGGGCCTTTCTGCGTTTATACGGAGACACTTTATGTTTCGACTTCATTACAACAAAAGCtt                                                                                                                                                                                                                                                                              >  1:257474/1‑299`  
                                         `ATGGATGAGTTCACTGAGACAGTAAAAGAGGCTATCCTAAGTCAATGGGGATTCTTTAGCCCATACGGTATTGGCGACAACGATGCTTGTACTATTAACCCTTACGATCGCTGGTCGTGTAAAATTATCTACATGGAGGAAACACCTGATGGCTCTTTGCGGCACCCATCGTTCGTAATGTTCTGTGGCACCGAGGTCAACCATCACGAGAAAATGTAATAACTCTGGCTCACCTTCGGGTGGGCCTTTCTGCGTTTATAAGGAGATACTATATGgctcctatacacattctcgagcccac                                                                                                                                                                                                                                                                              >  1:549252/1‑275`  
                                                                                                           `GGTATTGGCGACAACGATGCTTGTACTATTAACCATTCCGATGGCTGGGCGTGTCAAATTAGCTACATGGAGGAAACCCCTGATGGCTCTTTGCGGCACCCATCGTTCGTAATGTTCCGTGGCACCGAGGACAACCCTCAAGAGAAAATGTAATCACACTGGCTCACCTTCGGGTGGGCCTTTCTGCGTTTATAAGGAGACACTTTATGTTTCGACTTCATTACAACAAAAGCGTTAAGAATTTCACGGTTCGCCGTGCTGACCGTTCAATCGTATGTGCGAGCGAGCGCCGAGCTAAGAT                                                                                                                                                                                                            <  1:731358/301‑1`  
                                                                                                                    `gggctcggagatgtgtataagagacagTACGATGGCTGGGCGTGTCAAATTAGCTACATGGAGGAAACACCTGATGGCTCTTTGCGGCACCCATCATTCGTAATGTTCCGTGGCACCGAGGACAACCCTAAAGAGAAAATGTAATCACACTGGCTCACCTTCGGGTGGGCCTTTCTGCGTTTATAAGGAGACACTTTATGTTTCGACTTCATTACAACAAAAGCGTTAAGAATTTCACGGTTCGCCGTGCTGACCGTTCAATCGTATGTGCGAGCGAGCGCCGAGCTAAGATACCTCTTAT                                                                                                                                                                                                   <  1:67744/274‑1`  
                                                                                                                        `ataagagacagACTATTAACCCTTACGATGGCTGGGCGTGTCAAATTAGCTACATGGAGGAAACACCTGATGGCTCTTTGCGGCACCCATCGTTCGTAATGTTCCGTGGCACCGAGGACAACCCTCAAGAGAAAATGTAATCACACTGGCTCACCTTCGGGTGGGCCTTTCTGCGTTTATAAGGAGACACTATATGTTTCGACTTCATTACAACAAAAGCGTTAAGAATTTCACGGTTCGCCGTGCTGACCGTTCAATCGTATGTGCGAGCGAGCGCCGAGCTAAGATACCTCTTATTGGT                                                                                                                                                                                               <  1:500115/290‑1`  
                                                                                                                                      `tgtataagagacagTGGCTGGGCGTGTCAAATTAGCTACATGGAGGAAACACCTTATTGCTCTTTGCGGCACCCATCGTTCGTAATGTTCCGTGGCACCGAGGACAACCCTCAAGAGAAAATGTAATCACACTGGCTCACCTTCGTGTGGGCCTTTCTGCGTTTATAAGGAGACACTTTATGTTTCGACTTCATTACAACAAAAGCGTTAAGAATTTCACGGTTCGCCGTGCTGACCGTTCAATCGTATGTGCGAGCGAGCGCCGAGCTAAGATACCTCTTATTGGTAACACAGTTCCTTT                                                                                                                                                                                 <  1:388889/287‑1`  
                                                                                                                                                                                                                                         `GAGGACAACCCTCAAGAGAAAATGTAATCACACTGGCTCACCTTCGGGTTGGCCTTTCTGCGTTTATAAGGAGACACTATATGTTTCGACTTCATTACAACAAAAGCGTTAAGAATTTCACGGTTCGCCGTGCTGACCGTTCAATCGTATGTGCGAGCGAGCGCCGAGCTAAGATACCTCTTATTTGTAACACAGTTCCTTTGGCACCTAGCGTCCACATCATTATCACCCGTGGTGACTTTGAGAAAGCAATAGACAAGAAACGTCCGGTTCTTAGTGTGGCctgtctcttatacacatc                                                                              >  1:713407/1‑283`  
                                                                                                                                                                                                                                                                           `GGCTCACCTTCGGGTGGGCCTTTCTGCGTTTATAATGAAACACTATATGTTTCGACTTCATTACAACAAAAGCGTTAAGAATTTCACGGTTCGCCGTGCTGACCGTTCAATCGTATGTGCGAGCGAGCGCCGAGCTAAGATACCTCTTATTGGTAACACAGTTCCTTTGGCACCGAGCGTCCACATCATTATCACCCGTGGTGACTTTGAGAAAGTAATAGACAAGAAACGTCCGGTTCTTAGTGTGACAGTGACCCGCTTCCCGTTCGTCCGTCTGTTACTCAAACGAATCAAGGAGGTG                                            >  1:316472/1‑301`  
                                                                                                                                                                                                                                                                                              `gatgtgtataagagacaGAGACACTATATGTTTCGACTTCATTACAACAAAAGCGTTAAGAATTTCACGGTTCGCCGTGCTGACCGTTCAATCGTATGTGCGAGCGAGCGCCGAGCTAAGATACCTCTTATTGGTAACACAGTTCCTTTGGCACCGAGCGTCCACATCATTATCACCCGTGGTGACTTTGAGAAAGCAATAGACAAGAAACGTCCGGTTCTTAGTGTGGCAGTGACCCGCTTCCCGTTCGTACGTCTGTTACTCAAACGAATCAAGGAGGTGTTCTGATGGGACTGTTAGA                         <  1:593249/284‑1`  
                                                                                                                                                                                                                                                                                               `gagatgtgtataagagacagCACTATATGTTTCGACTTCATTACAACAAAAGCGTTAAGAATTTCACGGTTCGCCGTGCTGACCGTTCAATCGTATGTGCGAGCGAGCGCCGAGCTAAGATACCTCTTATTGGTAACACAGTTCCCTTTGCACCGAGCGTCCAAATCATTATCACCCGTGGTGACTTTGAGAAAGCAATAGACAAGAAACGTCCGGTTCTTAGTGTGGCAGTGACCCGCTTCCCGTTCGTCCGTCTGTTACTCAAACGAATCAAGGAGGTGTTCTGATGGGACTGTTAGAT                        <  1:367944/281‑1`  
                                                                                                                                                                                                                                                                                                 `ccgcctaacttaagtgGACACTATATGTTTCGACTTCATTGCAACACATGCGTTCAGAATGTCGCGGTTCGCCGTTCTGACCATTCCATCGTATGTGCGAGCGGGCGCCGAGCTAAGATTCCTCTTTTTGGTAACACAGTTCCTTTGGCACCGAGCGTCCACATCATTATCACCCGTGGTGACTTTTAGAAAGCAATAGACAAGAAACGTCCGGTTCTTAGTGTGGCAGTGACCCGCTTCCCGTTCGTCAGTCTGTTACTCAAACGAATCAAGGAGGTGTTCTGATGGGACTGTTAGATGG                      <  1:40072/285‑1`  
                                                                                                                                                                                                                                                                                                         `ctacAGGATACACTATATGTTTCGACTTCATTACAACAAAAGCGTTAAGAATTTCACGGTTCGCCGTGCTGACCGTTCAATCGTATGTGCGAGCGAGCGCCGAGCTAAGATACCTCTTATTGGTAACACAGTTCCTTTGGCACCGAGCGTCCACATCATTATCACCCGTGGTGACTTTGAGAAAGCAATAGACAAGAAACGTCCGGTTCTTAGTGTGGCAGTGACCCGCTTCCCGTTCGTCCGTCTGTTACTCAAACGAATCAAGGAGGTGTTCTGATGGGACTGTTAGATGGTGAAGCCT              >  1:157804/5‑301`  
                                                                                                                                                                                                                                                                                                             `caGAGACACTATATGTTTCGACTTCATTACAACAAAAGCGTTAAGAATTTCACGGTTCGCCGTGCTGACCGTTCAATCGTATGTGCGAGCGAGCGCCGAGCTAAGATACCTCTTATTGGTAACACAGTTCCTTTGGCACCGAGCGTCCACATCATTATCACCCGTGGCGACTTTGAGAAAGCAATAGACAAGAAACGTCCGGTTCTTAGTGTGGCAGTGACCCGCTTCCCGTTCGTCCGTCTGTTACTCAAACGAATCAAGGAGGTGTTCTGATGGGACTGTTAGATGGTGAAGCCTGGGA          <  1:733740/299‑1`  
                                                                                                                                                                                                                                                                                                             `agacagCACTATATGTTTCGACTTCATTACAACAAAAGCGTTAAGAATTTCACGGTTCTCCGTGCTGACCGTTCAATCGTATGTGCGAGCGAGCGCCGAGCTAAGATACCTCTTATTGGTAACACAGTTCCTTTGGCACCGAGCGTCCACATCATTATCACCCGTGGTGACTTTGAGAAAGCAATAGACAAGAAACGTCCGGTTCTTAGTGTGGCAGTGACCCGCTTCCCGTTCGTCCGTCTGTTACTCAAACGAATCAAGGAGGTGTTCTGATGGGACTGTTAGATGGTGAAGCCTGGGA          <  1:482905/295‑1`  
                                                                                                                                                                                                                                                                                                             `AGGAGACACTATATGTTTCGACTTCATTACAACAAAAGCGTTAAGAATTTCACGGTTCGCCGTGCTGACCGTTCAATCGTATGTGCGAGCGAGCGCTGAGCTAAGATACCTCTTATTGGTAACACAGTTCCTTTGGCACCGAGCGTCCACATCATTATCACTCGTGGTGACTTTGAGAAAGCAATAGACAAGAAACGTCCGGTTCTTAGTGTGGCAGTGACCCGCTTCCCGTTCGTCCGTCTGTTACTCAAACGAATCAATGAGGTGTTCTGATGGGACTGTTAGATGGTGAAGCCTGGGA          >  1:110285/1‑301`  
                                                                                                                                                                                                                                                                                                             `AGGAGACACTATATGTTTCGACTTCATTACAACAAAAGCGTTAAGAATTTCACGGTTCGCCGTGCTGACCGTTCAATCGTATGTGCGAGCGAGCGCCGAGCTAAGATACCTCTTATTGGTAACACAGTTCCTTTGGCACCGAGCGTCCACATCATTATCACCCGTGGTGACTTTGAGAAAGCAATAGACAAGAAACGTCCGGTTCTTAGTGTGGCAGTGACCCGCTTCCCGTTCGTCCGTCTGTTACTCAAACGAATCAAGGAGGTGTTCTGATGGGACTGTTAGATGGTGAAGCCTGGGA          >  1:257979/1‑301`  
                                                                                                                                                                                                                                                                                                             `AGGAGACACTATATGTTTCGACTTCATTACAACAAAAGCGTTAAGAATTTCACGGTTCGCCGTGCTGACCGTTCAATCGTATGTGCGAGCGAGCGCCGAGCTAAGATACCTATTATTGGTAACACAGTTCCTTTGGCACCGAGCGTCCACATCATTATCACCCGTGGTGACTTTGAGAAAGCAATAGACAAGAAACGTCCGGTTCTTAGTGTGGCAGTGACCCGCTTCCCGTTCGTCCGTCTGTTACTCAAACGAATCAAGGAGGTGTTCTGATGGGACTGTTAGATGGTGAAGCCTGGGA          <  1:729894/301‑1`  
                                                                                                                                                                                                                                                                                                             `AGGAGACACTAAATGTTTCGACTTCATTACAACAAAAGCGTTAAGAATTTCACGGTTCGCCGTGCTGACCGTTCAATCGTATGTGCGAGCGAGCGCCGAGCTAAGATACCTCTTATTGGTAACACAGTTCCTTTGGCACCGAGCGTCCACATCATTATCACCCGTGGTGACTTTGAGAAAGCAATAGACAAGAAACGTCCGGTTCTTAGTGTGGAAGTGACCCGCTTCCCGTTCGTCCGTCTGTTACTCAAACGAATCAAGGAGGTGTTCTGATGGGACTGTTAGATGGTGAAGCCTGGGA          <  1:392826/301‑1`  
                                                                                                                                                                                                                                                                                                              `aGAGACACTATATGTTTCGACTTCATTACAACAAAAGCGTTAAGAATTTCACGGTTCGCCGTGCTGACCGTTCAATCGTATGTGCGAGCGAGCGCCGAGCTAAGATACCTCTTATTGGTAACACAGTTCCTTTGGCACCGAGCGTCCACATCATTATCACCCGTGGTGACTTTGAGAAAGCAATAGACAAGAAACGTCAGGTTCTTAGTGTGGCAGTGACCCGCTTCCCGTTCGTCCGTCTGTTACTCAAACGAATCAAGGAGGTGTTCTGATGGGACTGTTAGATGGTGAAGCCTGGGAA         <  1:365205/300‑1`  
                                                                                                                                                                                                                                                                                                              `aGAGACACTATATGTTTCGACTTAATTACAACAAAAGCGTTAAGAATTTCACGGTTCGCCGTGCTGACCGTTCAATCGTATGTGCGAGCGAGCGCCGAGCTAAGATACCTCTTATTGGTAACACAGTTCCTTTGGCACCGAGCGTCCACATCATTATCACCAGTGGTGACTTTGAGAAAGCAATAGACAAGAAACGTCCGGTTCTTAGTGTGGCAGTGACCCGCTTCCCGTTCGTCCGTCTGTTACTCAAACGAATAAAGGAGGTGTTCTGATGGGACTGTTAGATGGTGAAGCCTGGGAA         <  1:730791/300‑1`  
                                                                                                                                                                                                                                                                                                              `GGAGACACTATATGTTTCGACTTCATTACAACAAAAGCGTTAAGAATTTCACGGTTCGCCGTGCTGACCGTTCAATCGTATGTGCGAGCGAGCGCCGAGCTAAGATACCTCTTATTTGTAACACAGTTCCTTTGGCACCGAGCGTCCACATCATTATCACCCGTGGTGACTTTGAGAAAGCAATAGACAAGAAACGTCCGGTTCTTAGTGTGGCAGTGACCCGCTTCCCGTTCGTCCGTCTGTTACTCAAACGAATCAAGGAGGTGTTCTGATGGGACTGTTAGATGGTGAAGCCTGGGAA         >  1:111460/1‑301`  
                                                                                                                                                                                                                                                                                                              `GGAGACACTATATGTTTCGACTTCATTACAACAAAAGCGTTAAGAATTTCACGGTTCGCCGTGCTGACCGTTCAATCGTATGTGCGAGCGAGCGCCGAGCTAAGATACCTATTATTGGTAACACAGTTCCTTTGGCACCGAGCGTCCACATCATTATCACCCGTGGTGACTTTGAGAAAGCAATAGACAAGAAACGTCCGGTTCTTAGTGTGGCAGTGACCCGCTTCCCGTTCGTCCGTCTGTTACTCAAACGAATCAAGGAGGTGTTCTGATactgtctcttatacacatctccgagccc         >  1:35859/1‑273`  
                                                                                                                                                                                                                                                                                                                  `gCACTATATGTTTCGACTTCATTACAACAAAAGCGTTAAGAATTTCACGGTTCGCCGTGCTGACCGTTCAATCGTATGTGCGAGCGAGCGCCGAGCTAAGATACCTCTTATTGGTAACACAGTTCCTTTGGCACCGAGCGTCCACATCATTATCACCTGTGGTGACTTTGAGAAAGCAATAGACAAGAAACGTCCTGTTCTTAGTGTGGCAGTGACCCGCTTCCCGTTCGTCCGTCTGTTACTCAAACGAATCAAGTAGGTGTTCTGATGGGACTGTTAGATGGTGAAGCCTGGGAAAAAG     >  1:302058/2‑301`  
                                                                                                                                                                                                                                                                                                                  `ACACTATATGTTTCGACTTCATTACAACAAAAGCGTTAAGAATTTCACGGTTCGCCGTGCTGACCGTTCAATCGTATGTGCGAGCGAGCGCCGAGCTAAGATACCTCTTATTGGTAACACAGTTCCTTTGGCACCGAGCGTCCACATCATTATCACCCGTGGTGACTTTGAGAAAGCAATAGACAAGAAACGTCAGGTTCTTAGTGTGGCAGTGACCCGCTTCCCGTTCGTCCGTCTGTTACTCAAACGAATCAAGGAGGTGTTCTGATGTGACTGTTAGATGGTGAAGCCTGGGAAAAAG     >  1:425083/1‑301`  
                                                                                                                                                                                                                                                                                                                   `CACTATATTTTTCGACTTCATTACAACAAAAGCGTTAAGAATTTCACGGTTCGCCGTGCTGACCGTTCAATCGTATGTGCGAGCGAGCGCCGAGCTAAGATACCTCTTATTGGTAACACAGTTCCTTTGGCACCGAGCGTCCACATCATTATCACCCGTGGTGACTTTGAGAAAGCAATAGACAAGAAACGTCCGGTTCTTAGTGTGGCAGTGACCCGCTTCCCGTTCGTCCGTCTGTTACTCAAACGAATCAAGGAGGTGTTCTGATGGGACTGTTAGATGGTGAAGCCTGGGAAAAAGA    >  1:545734/1‑301`  
                                                                                                                                                                                                                                                                                                                   `CACTATATGTTTCGACTTCATTACAACAAAAGCGTTAAGAATTTCACGGTTCGCCGTGCTGACCGTTCAATCGTATGTGCGAGCGAGCGCCGAGCTAAGATACCTCTTATTGGTAACACAGTTCCTTTGGCACCGAGCGTCCACATCATTATCACCCGTGGTGACTTTGAGAAAGCAATAGACAAGAAACGTCCGGTTCTTAGTGTGGCAGTGACCCGCTTCCCGTTCGTCCGTCTGTTACTCAAACGAATCAAGGAGGTGTTCTGATGGGACTGTTAGATGGctgactcttatacacatc    >  1:613135/1‑283`  
                                                                                                                                                                                                                                                                                                                   `CACTATATGTTTCGACTTCATTACAACAAAAGCGTTAAGAATTTCACGGTTCGCCGTGCTGACCGTTCAATCGTATGTGCGAGCGAGCGCCGAGCTAAGATACCTCTTATTGGTAACACAGTTCCTTTGGCACCGAGCGTCCACATCATTATCACCCGTGGTGACTTTGAGAAAGCAATAGACAAGAAACGTCCGGTTCTTAGTGTAGCAGTGACCCGCTTCCCGTTCGTCCGTATGTTACTCACAAGAATCAAGGAGGTGTTCTGATGGGACTGTTAGATGGTGAAGCCTGGGAAAAAGA    >  1:187746/1‑301`  
                                                                                                                                                                                                                                                                                                                   `CACTATATGTTTCGACTTCATTACAACAAAAGCGTTAAGAATTTCACGGTTCGCCGTGCTGACCGTTCAATCGTATGTGCGAGCGAGCGCCGAGCTAAGATACCTCTTATTAGTAACACAGTTCCTTTGGCACCGAGCGTCCACATCATTATCACCCGTGGTGACTTTGAGAAAGCAATAGACAAGAAACGTCCGGTTCTTAGTGTGGAAGTGACCCGCTTCCCGTTCGTCCGTATGTTACTCAAACGAATCAAGGAGGTGTTCTGATGGGACTGTTAGATGGTGAAGCCTGGGAAAAAGA    >  1:331118/1‑301`  
                                                                                                                                                                                                                                                                                                                   `CACTATATGTTTCGACTTCATTACAACAAAAGCGTTAAGAATTTCACGGTTCGCCGTGCTGACCGTTCAATCGTATGTGCGAGCGAGCGCCGAGATAAGATACCTCTTATTGGTAACACAGTTCCTTTGGCACCGAGCGTCCACATCATTATCACCCGTGGTGACTTTGAGAAAGCAATAGACAAGAAACGTCCGGTTCTTAGTGTGGCAGTGACCCGCTTCCCGTTCGTCCGTCTGTTACTCAAACGAATCAAGGAGGTGTTCTGATGGGACTGTTAGATGGTGAAGCCTGGGAACAAGA    >  1:727914/1‑301`  
                                                                                                                                                                                                                                                                                                                   `CACTATATGTTTCGACTTCATTACAACAAAAGCGTTAAGAATTTCACGGTTCGCCGTGCTGACCATTCAATCGTATGTGCGAGCGAGCGCCGAGCTAAGATACCTCTTATTGGTAACACAGTTCCTTTGGCACCGAGCGTCCACATCATTATCACACGTGGTGACTTTGAGAAAGCAATAGACAAGAAACGTCCGGTTCTTAGTGTGGCAGTGACCCGCTTCCCGTTCGTCCGTCTGTTACTCAAACGAATCAAGGAGGTGTTCTGATGGGctgtctcttatacacatctccgagcccacg    >  1:183047/1‑271`  
                                                                                                                                                                                                                                                                                                                     `CTATATGTTTCGACTTCATTACAACAAAAGCGTTAAGAATTTCACGGTTCGCCGTGCTGACCGTTCAATCGTATGTGCGAGCGAGCGCCGAGCTAAGATACCTCTTATTGGTAACACAGTTCCTTTGGCACCGAGCGTCCACATCATTATCACCCGTGGTGACTTTGAGAAAGCAATAGACAAGAAACGTCCGGTTCTTAGTGTTGCAGTGACCCGCTTTCCGTTCGTCCGTCTGTTACTCACACGAATCAAGGATGTGTTCTGATGGGACTGTTAGATctgtctcttatacacatctccg  >  1:541053/1‑279`  
                                                                                                                                                                                                                                                                                                                     `CTATATGTTTCGACTTCATTACAACAAAAGCGTTAAGAATTTCACGGTTCGCCGTGCTGACCGTTCAATCGTATGTGCGAGCGAGCGCCGAGCTAAGATACCTCTTATTGGTAACACAGTTCCTTTGGCACCGAGCGTCCACATCATTATCACCCGTGGTGACTTTGAGAAAGCAATAGACAAGAAACGTCCGGTTCTTAGTGTGGCAGTGACCCGCTTCCCGTTCGTCCGTCTGTTACTCAAACGAATCAAGGAGGTGTTCTGATGGGACctgtctcttatacacatctccgagcccacg  >  1:157877/1‑271`  
                                                                                                                                                                                                                                                                                                                     `CTATATGTTTCGACTTCATTACAACAAAAGCGTTAAGAATTTCACGGTTCGCCGTGCTGACCGTTCAATCGTATGTGCGAGCGAGCGCCGAGCTAAGATACCTCTTATTGGTAACACAGTTCCTTTGGCACCGAGCGTCCACATCATTATCACCCGTGGTGACTTTGAGAAAGCAATAGACAAGAAACGTCCGGTTCTTAGTGTGGCAGTGACCCGCTTCCCGTTCGTCCGTCTGTTACTCAAACGAATCAAGGAGGTGTTCTGATGGGACTGTTAGATGGTGAAGCCTGGGAAAAAGAAA  >  1:568096/1‑301`  
                                                                                                                                                                                                                                                                                                                     `CTATATGTTTCGACTTCATTACAACAAAAGCGTTAAGAATTTCACGGTTCGCCGTGCTGACCGTTAAATCGTATGTGCGAGCGAGCGCCGAGCTAAGATACCTCTTATTGGTAACACAGTTCCTTTGGCACCGAGCGTCCACATCATTATCACCCGTGGTGCCTTTGAGAAAGACATAGACAAGAAACGTCCGGTTCTTAGTGTGGCAGTGACCCGCTTCCCGTTCGTCCGTCTGTTACTCAAACGAATCAAGGAGGTGTTCTGATGGACCTGTTAGATGGTGAAGCCTGGGAActgtcct  >  1:135700/1‑294`  
                                                                                                                                                                                                                                                                                                                     `CTATATGTTTCGACTTCATTACAACAAAAGCGTTAAGAATTTCACGGTTCGCCGTGCTGACCATTCAATCGTATGTGCGAGCGAGCGCCGAGCTAAGATACCTCTTATTGGTAACACAGTTCCTTTGGCACCGAGCGTCCACATCATTATCACCCGTGGTGACTTTGAGAAAGCAATAGACAAGAAACGTACGGTTCTTAGTGTGGCAGTGACCCGCTTCCCGTTCGTCCGTCTGTTACTCAAACAAATCAAGGAGGTGTTCTGATGGGACTGTTAGATctgtctcttatacacatctccg  >  1:100640/1‑279`  

                                                                                                                                                                                                                                                                                                                                                                                                                                                                                                                                                                                                                                    
`GTGGTCGTTTAGTTAACGCCACGAATATCTCTCGCGCCTTAATGGATGAGTTCACTGAGACAGTAAAAGAGGCCACCCTAAGTCAATGGGGATTCTTTAGCCCATACGGTATTGGCGACAACGATGCTTGTACTATTAACCCTTACGATGGCTGGGCGTGTCAAATTAGCTACATGGAGGAAACACCTGATGGCTCTTTGCGGCACCCATCGTTCGTAATGTTCCGTGGCACCGAGGACAACCCTCAAGAGAAAATGTAATCACACTGGCTCACCTTCGGGTGGGCCTTTCTGCGTTTATAAGGAGACACT‑‑‑‑‑‑‑‑‑‑‑‑‑‑‑‑‑‑‑‑‑‑‑‑‑‑‑‑‑‑‑‑‑‑‑‑‑‑‑‑‑‑‑‑‑‑‑‑‑‑‑‑‑‑‑‑‑‑‑‑‑‑‑‑‑‑‑‑‑‑‑‑‑‑‑‑‑‑‑‑‑‑‑‑‑‑‑‑‑‑‑‑‑‑‑‑‑‑‑‑‑‑‑‑‑‑‑‑‑‑‑‑‑‑‑‑‑‑‑‑‑‑‑‑‑‑‑‑‑‑‑‑‑‑‑‑‑‑‑‑‑‑‑‑‑‑‑‑‑‑‑‑‑‑‑‑‑‑‑‑‑‑‑‑‑‑‑‑‑‑‑‑‑‑‑‑‑‑‑‑‑‑‑‑‑‑‑‑‑‑‑‑‑‑‑‑‑‑‑‑‑‑‑‑‑‑‑‑‑‑‑‑‑‑‑‑‑‑‑‑‑‑‑‑‑‑‑‑‑‑‑‑‑‑‑‑‑‑‑‑‑‑‑‑‑‑‑‑‑‑‑‑‑‑‑‑‑‑‑‑‑‑‑‑‑‑‑‑‑‑‑‑‑‑‑‑‑‑‑‑‑‑‑‑‑‑‑‑‑‑‑‑‑‑‑‑‑‑‑  >  NC_001604.1/7295‑7605`  
`‑‑‑‑‑‑‑‑‑‑‑‑‑‑‑‑‑‑‑‑‑‑‑‑‑‑‑‑‑‑‑‑‑‑‑‑‑‑‑‑‑‑‑‑‑‑‑‑‑‑‑‑‑‑‑‑‑‑‑‑‑‑‑‑‑‑‑‑‑‑‑‑‑‑‑‑‑‑‑‑‑‑‑‑‑‑‑‑‑‑‑‑‑‑‑‑‑‑‑‑‑‑‑‑‑‑‑‑‑‑‑‑‑‑‑‑‑‑‑‑‑‑‑‑‑‑‑‑‑‑‑‑‑‑‑‑‑‑‑‑‑‑‑‑‑‑‑‑‑‑‑‑‑‑‑‑‑‑‑‑‑‑‑‑‑‑‑‑‑‑‑‑‑‑‑‑‑‑‑‑‑‑‑‑‑‑‑‑‑‑‑‑‑‑‑‑‑‑‑‑‑‑‑‑‑‑‑‑‑‑‑‑‑‑‑‑‑‑‑‑‑‑‑‑‑‑‑‑‑‑‑‑‑‑‑‑‑‑‑‑‑‑‑‑‑‑‑‑‑‑‑‑‑‑‑‑‑‑‑‑‑‑‑‑‑‑‑‑‑‑‑‑‑‑‑‑‑‑‑‑‑‑‑‑‑‑‑‑‑‑‑‑‑‑‑‑‑‑‑‑aaggagacactATATGTTTCGACTTCATTACAACAAAAGCGTTAAGAATTTCACGGTTCGCCGTGCTGACCGTTCAATCGTATGTGCGAGCGAGCGCCGAGCTAAGATACCTCTTATTGGTAACACAGTTCCTTTGGCACCGAGCGTCCACATCATTATCACCCGTGGTGACTTTGAGAAAGCAATAGACAAGAAACGTCCGGTTCTTAGTGTGGCAGTGACCCGCTTCCCGTTCGTCCGTCTGTTACTCAAACGAATCAAGGAGGTGTTCTGATGGGACTGTTAGATGGTGAAGCCTGGGAAAAAGAAA  >  NC_001604.1/7904‑8202`

  

Alignment Legend

Aligned base mismatch/match (shaded by quality score): `ATCG/ATCG` `< 3 ≤` `ATCG/ATCG` `< 18 ≤` `ATCG/ATCG` `< 29 ≤` `ATCG/ATCG` `< 37 ≤` `ATCG/ATCG` `< 38 ≤` `ATCG/ATCG`

Unaligned base: `atcg`    Masked matching base: `atcg`    Alignment gap: `‑`    Deleted base: `‑`

Reads not counted as support for junction

read\_name Not counted due to insufficient overlap past the breakpoint.

read\_name Not counted due to not crossing MOB target site duplication.
